# Inhibition of the MDM2 E3 Ligase Induces Apoptosis and Autophagy in Wild-Type and Mutant p53 Models of Multiple Myeloma, and Acts Synergistically with ABT-737

**DOI:** 10.1371/journal.pone.0103015

**Published:** 2014-09-02

**Authors:** Dongmin Gu, Shuhong Wang, Isere Kuiatse, Hua Wang, Jin He, Yun Dai, Richard J. Jones, Chad C. Bjorklund, Jing Yang, Steven Grant, Robert Z. Orlowski

**Affiliations:** 1 Department of Lymphoma/Myeloma, The University of Texas MD Anderson Cancer Center, Houston, Texas, United States of America; 2 Department of Hematology, Chinese PLA General Hospital, Beijing, China; 3 Department of Medicine, Virginia Commonwealth University, Richmond, Virginia, United States of America; 4 Department of Experimental Therapeutics, The University of Texas MD Anderson Cancer Center, Houston, Texas, United States of America; Rush University Medical Center, United States of America

## Abstract

Intracellular proteolytic pathways have been validated as rational targets in multiple myeloma with the approval of two proteasome inhibitors in this disease, and with the finding that immunomodulatory agents work through an E3 ubiquitin ligase containing Cereblon. Another E3 ligase that could be a rational target is the murine double minute (MDM) 2 protein, which plays a role in p53 turnover. A novel inhibitor of this complex, MI-63, was found to induce apoptosis in p53 wild-type myeloma models in association with activation of a p53-mediated cell death program. MI-63 overcame adhesion-mediated drug resistance, showed anti-tumor activity *in vivo*, enhanced the activity of bortezomib and lenalidomide, and also overcame lenalidomide resistance. In mutant p53 models, inhibition of MDM2 with MI-63 also activated apoptosis, albeit at higher concentrations, and this was associated with activation of autophagy. When MI-63 was combined with the BH3 mimetic ABT-737, enhanced activity was seen in both wild-type and mutant p53 models. Finally, this regimen showed efficacy against primary plasma cells from patients with newly diagnosed and relapsed/refractory myeloma. These findings support the translation of novel MDM2 inhibitors both alone, and in combination with other novel agents, to the clinic for patients with multiple myeloma.

## Introduction

Multiple myeloma is a malignant plasma cell dyscrasia characterized clinically in patients with symptomatic disease by anemia, hypercalcemia, renal insufficiency, or bony lesions [1], [2], and is the second most commonly diagnosed hematologic malignancy [3]. Novel drug classes such as proteasome inhibitors and immunomodulatory agents have had a significant impact upon the natural history of this disease, with some studies suggesting a doubling in the median overall survival [4], [5], [6], [7], [8], [9]. Bortezomib and carfilzomib are the currently approved proteasome inhibitors for multiple myeloma, and exert their effects by blocking the turnover of poly-ubiquitinated proteins through the proteasome, which is the final effector of the ubiquitin-proteasome pathway [10], [11], [12]. Downstream effects of proteasome inhibition include activation of the endoplasmic reticulum stress response, inhibition of adherence and survival signaling through nuclear factor kappa B [10], [11], [12], and induction of a pro-apoptotic program, including through p53 [13], [14], [15], [16], [17]. Interestingly, recent studies of the immunomodulatory agents thalidomide, lenalidomide, and pomalidomide have indicated that they work in part through an effect on an E3 ubiquitin ligase that incorporates the immunomodulatory drug binding protein Cereblon [18], [19]. This influences substrate specificity of the ligase for targets such as the Ikaros and Aiolos transcription factors [20], [21], whose degradation results in anti-proliferative plasma cell effects and T cell stimulation.

Another E3 ubiquitin ligase that may be a rational target for multiple myeloma therapy is murine double minute (MDM) 2, a pleiotropic protein best known for facilitating the p53 ubiquitination required for its proteasome-mediated turnover [22]. Regulation of p53 function also occurs by binding of MDM2 to p53 amino acids 15–29, preventing p53 interactions with transcriptional machinery, forming a negative feedback loop limiting p53 accumulation and function [22]. MDM2 may be over-expressed in some cases of multiple myeloma [23], [24] through mechanisms such as gene amplification or chromosomal trisomy [24]. In addition, epigenetic suppression of the promoter for the micro RNA 194-2-192 cluster may also enhance MDM2 expression [25]. This over-expression has been shown to result in enhanced cell cycle progression, proliferation, and survival of myeloma cells, in part through down-regulation of the cyclin-dependent kinase inhibitor p21 [26]. Since p21 accumulation is a part of the mechanism of action for both proteasome inhibitors [14] and immunomodulatory agents [19], [27], these findings together support the possibility that approaches targeting MDM2 could be attractive options for myeloma patients.

Small molecule inhibitors of the MDM2/p53 interaction have been identified, such as the Nutlins [28], which were the first generation of agents directly targeting this pathway, and bind in MDM2's p53 binding pocket in the N-terminal region to induce p53 accumulation. These have helped in elucidating the biology of MDM2 and p53, such as by allowing identification of new MDM2 targets [29], and have shown pre-clinical activity against myeloma *in vitro* [30], [31], [32]. However, the low potency of these first generation agents has limited their potential clinical applicability. We therefore sought to determine if one of the second-generation MDM2 inhibitors [33], MI-63 [34], [35], could be active against myeloma, and if it could fit into our armamentarium in combination with other currently approved and novel agents against this disease. In this study, we present data which indicate that MI-63 has potent activity against myeloma using both *in vitro* and *in vivo* models. Also, studies in mutant p53 models show evidence of activity, and indicate activation of autophagy. Finally, MI-63 can overcome lenalidomide resistance, can be combined with other currently approved agents, such as bortezomib or lenalidomide, and also with novel drugs including the BH3 mimetic ABT-737, to enhance activity against both myeloma cell lines and primary samples. Together, the data support the translation of approaches targeting the interaction between MDM2 and p53 to the clinic for patients with relapsed and/or refractory myeloma.

## Materials and Methods

### Reagents

MI-63 and MI-219 were provided by Sanofi (Bridgewater, NJ), while ABT-737, bortezomib, and lenalidomide were purchased from Selleck Chemicals (Houston, TX).

Chloroquine and 3-methyladenine were purchased from Sigma-Aldrich (St. Louis, MO).

### Tissue culture and patient samples

Myeloma cell lines were purchased either from the German Collection of Microorganisms and Cell Cultures (Braunschweig, Germany), or the American Type Culture Collection (Manassas, VA), and validated by the MD Anderson Characterized Cell Line Core Facility. Primary samples were from patients who had provided written informed consent in compliance with the Declaration of Helsinki according to an MD Anderson Institutional Review Board 5 approved protocol (LAB11-0321). CD138^+^ or ^−^ cells were isolated from these fresh bone marrow aspirates with the CD138 Positive Plasma Cell Isolation Kit (Miltenyi Biotec; Auburn, CA). Cells were cultured in RPMI 1640 medium with 2 mM L-glutamine (Invitrogen; Carlsbad, CA) supplemented with 10% fetal bovine serum (Sigma-Aldrich), 100 U/mL penicillin (Invitrogen) and 100 µg/ml streptomycin (Invitrogen). HS-5 stromal cells from the American Type Culture Collection were cultured in Dulbecco's modified Eagle's medium containing fetal bovine serum and penicillin and streptomycin as above.

### Cell viability assays

Cell viability was determined using the tetrazolium reagent WST-1 (Roche Applied Science; Indianapolis, IN) according to the manufacturer's instructions and as previously described [36]. Viability curves were fitted in GraphPad Prism version 6 (La Jolla, CA) and median inhibitory concentrations (IC_50_) were calculated using log (inhibitor) vs. response – variable slope (four parameters).

### shRNA gene knockdown

Lentiviral constructs containing non-targeting shRNA sequences, or shRNAs designed to suppress expression of MDM2, p53, autophagy (ATG)-related protein 5 (ATG5) and Beclin-1 were purchased from Sigma-Aldrich. Viral particles were generated from 293T cells following standard protocols, and myeloma cells were infected and selected with the use of polybrene and puromycin, as detailed previously [37].

### Reverse transcription and quantitative PCR

Total RNA was extracted using Trizol (Invitrogen), and cDNA was synthesized with High-Capacity cDNA Reverse Transcription Kits (Applied Biosystems; Grand Island, NY) as previously described [38]. TaqMan Gene Expression Master Mix and probes were purchased from Applied Biosystems and used to perform quantitative PCR (qPCR) reactions on an Applied Biosystems StepOnePlus Real-Time PCR system. Expression of glyceraldehyde 3-phosphate dehydrogenase (GAPDH) was used as an internal control.

### Proteomic assays

Western blotting and immunoprecipitation of protein extracts was performed using standard procedures [39]. Antibodies which were used included: anti-p53 (DO-1) and Bax (6A7)(Santa Cruz Biotechnology; Santa Cruz, CA); anti-MDM2 (Ab-1) and -Bak (Ab-1)(Calbiochem; San Diego, CA); anti-Caspase-3 (5A1E), -9 (D2D4), -poly ADP ribose polymerase (PARP)(D64E10), -p53 upregulated modulator of apoptosis (PUMA)(D30C10), -Microtubule-associated protein 1 light chain 3 (LC3)(D3U4C & D11), -Cytochrome C (136F3), -Beclin-1 (#3738) and -ATG5 (#2630)(Cell Signaling Technology; Danvers, MA); and anti-Actin (A2066)(Sigma-Aldrich). Densitometry was performed using ImageJ software version 1.46 (National Institute of Health; Bethesda, MD). Mitochondrial isolation prior to Western blotting was performed where indicated using the Mitochondria Isolation Kit (Thermo Scientific; Rockford, IL). Reverse phase protein array (RPPA) analyses were performed by the MD Anderson Cancer Center RPPA/Functional Proteomics Core Facility.

### Cell cycle analysis and apoptosis

Cell cycle analysis was performed by staining with propidium iodide (Sigma-Aldrich), and then analyzing cells by flow cytometry as described previously [40]. Annexin V staining was used to detect apoptosis by flow cytometry using the manufacturer's instructions (Invitrogen).

### Drug synergy calculations and statistical analyses

Data were analyzed using CalcuSyn software (Biosoft; Cambridge, United Kingdom), and combination indices (CI) were calculated to determine if synergistic interactions were being observed. Statistical analyses were performed with unpaired *t* tests in GraphPad, and *p*-values less than 0.05 were judged to be significant.

### Xenograft model

Experiments were performed in accordance with procedures and protocols approved by the MD Anderson Cancer Center Animal Care and Use Committee using xenografts developed as detailed previously [36], [41]. In brief, 10^7^ MM1.S cells were inoculated subcutaneously in 6-week-old nonobese diabetic severe combined immunodeficiency (NOD/SCID) gamma mice (The Jackson Laboratory; Bar Harbor, ME) to establish tumors. Vehicle (10% polyethylene glycol, 3% Cremophor EL in phosphate buffered saline) or MI-219 (100 mg/kg) was injected intraperitoneally three times a week. Tumor diameters were measured with a digital caliper, and tumor volume was calculated by the formula: volume = (width)^2^×length/2.

## Results

### MDM2 inhibition with MI-63 is cytotoxic to wild-type p53 myeloma cells

**Figure 1:**
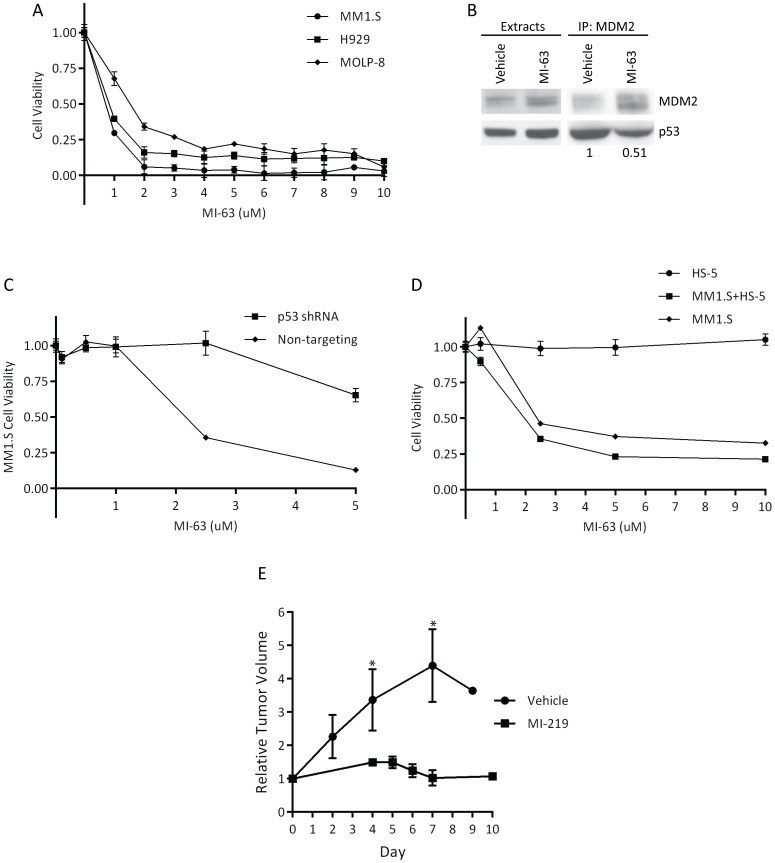
MI-63 is cytotoxic to wild-type p53 myeloma cells. **A.** Cell viability assays were performed in wild-type p53 myeloma cell lines exposed to the indicated concentrations of MI-63 for 48 hours using a tetrazolium reagent. Error bars represent standard errors of the mean from three or more replicates, and all experiments in this and later figures were repeated three times, with one representative figure shown. **B.** Whole cell extracts were prepared from MM1.S cells treated with vehicle or MI-63, and then subjected to immunoprecipitation with an antibody to MDM2. The precipitates were then probed by Western blotting using specific antibodies to MDM2 or p53, and densitometry was performed using Image J software. **C.** MM1.S cells stably infected with a Lentiviral vector expressing either a control, non-targeted shRNA or an shRNA directed at p53 were exposed to MI-63 as above, and viability was measured using the WST-1 reagent. **D.** Human stromal HS-5 cells or MM1.S myeloma cells were propagated in culture either alone, or co-cultured at a ratio of 20∶1, and viability was measured in the presence of MI-63. **E.** A murine xenograft myeloma model was developed in NOD/SCID mice, and when tumor volume reached 100 mm^3^, mice were treated intraperitoneally with 100 mg/kg MI-219 or vehicle, and tumor volume was monitored three times a week. Vehicle-treated mice experienced a substantial increase in the tumor burden, while MI-219 induced a significant tumor growth delay, (*p<0.05).

Nutlin-based inhibitors of MDM2 have been found to have activity predominantly against wild-type (wt) p53 myeloma models. To evaluate the activity of MI-63, we exposed a panel of wt p53 myeloma cell lines (MM1.S, H929, MOLP-8) to this agent, and measured viability with a tetrazolium reagent. All of these cell lines were found to be very sensitive to low drug levels over the course of a 48-hour exposure (Figure 1A), with a median inhibitory concentration (IC_50_) in the low single µM range or less (MM1.S: 0.6 µM; H929: 0.4 µM; MOLP-8: 1.5 µM). In order to verify that MI-63 was indeed inhibiting the interaction between MDM2 and p53, we subjected extracts of MM1.S cells to immunoprecipitation with an anti-MDM2 antibody, and then analyzed these by Western blotting. Compared to vehicle-treated controls, the level of p53 immunoprecipitated in association with MDM2 was reduced after these cells were treated with MI-63 (Figure 1B). In comparison, MI-63 did not alter the interaction with MDM4, which associates with MDM2 through the RING finger domain (Figure S1) [42]. We then prepared MM1.S cells in which p53 expression had been stably suppressed at the mRNA (Figure S2A) and protein levels (Figure S2B) using a Lentiviral-delivered small hairpin (sh) RNA construct. MI-63 retained activity against MM1.S cells harboring a non-targeting shRNA construct (Figure 1C), but this was substantially blunted in the cells with decreased p53 content (control shRNA IC_50_: 2.2 µM; p53 shRNA IC_50_: 5.2 µM), consistent with a strong impact of p53 status. Finally, since microenvironmental effects, such as expression of interleukin (IL)-6, may modulate the expression of p53 [43], we evaluated the activity of MI-63 on myeloma cells in the presence of HS-5 human stromal cells. When MM1.S cells were co-cultured with stromal cells, the IC_50_ of MI-63 was not significantly altered (MM1.S+HS-5 IC_50_: 1.9 µM; MM1.S IC_50_: 3.4 µM) (Figure 1D), and similar data were obtained in MOLP-8 cells (control shRNA IC_50_: 3.5 µM; p53 shRNA IC_50_: 3.5 µM; Figure S3). Next, we generated a xenograft model using NOD/SCID mice and MM1.S myeloma cells. Compared to vehicle-treated mice, which experienced a substantial increase in their tumor burden over the period of the experiment, treatment with the *in vivo* analogue of MI-63, MI-219, induced a significant tumor growth delay (Figure 1E).

### Molecular mechanisms of MI-63 action

**Figure 2:**
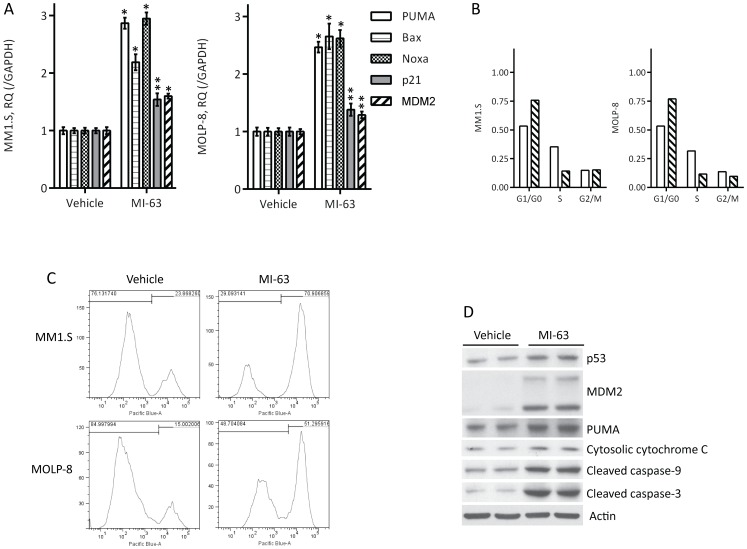
Molecular mechanisms underlying the action of MI-63. **A.** Quantitative PCR was performed of selected p53 transcriptional targets in wild-type cell lines after exposure to MI-63 at the IC_50_ for 48 hours. Values were normalized to GAPDH as a control (*p<0.005; **p<0.05). **B.** The indicated cell lines were treated with MI-63 at its IC_50_ for 48 hours, and cell cycle analysis was performed after propidium iodide staining. **C.** Activation of apoptosis was evaluated after Annexin V staining by flow cytometry in MM1.S and MOLP-8 cells treated with MI-63 for 48 hours. **D.** Western blotting of cells treated for 48 hours with MI-63 at the IC_50_ was performed to detect changes in key downstream targets, with Actin as the loading control.

Inhibition of MDM2 should result in accumulation of p53 protein with downstream activation of a p53-mediated cell death program, and we therefore examined these myeloma cell lines for evidence of this mechanism. Using qPCR, we found a significant increase of transcript levels for p53 up-regulated modulator of apoptosis (PUMA), Bcl-2 associated protein x (BAX), Noxa, p21, and MDM2 (Figure 2A). Many of these findings were then confirmed by RPPA, a high-throughput antibody-based technique developed for functional proteomics studies to evaluate protein activity in signaling networks. Results from MM1.S and MOLP-8 cells indicated an up-regulation of pro-apoptotic Bax, cell cycle regulators p21 and p27, and cleaved/active caspases-3 and -7, highlighting the activation of type I programmed cell death (Figure S4). Consistent in part with the induction of cyclin-dependent kinase inhibitors p21, p27, and with programmed cell death, cell cycle profiling showed an increased content of cells at G_0_/G_1_ after exposure to MI-63 (Figure 2B). Activation of apoptosis was confirmed using flow cytometry after Annexin V staining for cell surface phosphatidylserine levels, which were strongly enhanced by MDM2 inhibition (Figure 2C). Finally, at the protein level, MI-63 induced accumulation of p53, MDM2, and PUMA, and induced release of cytochrome c from mitochondria into the cytosol, resulting in activation of caspase-9 and the downstream effector caspase-3 (Figure 2D).

### Inhibition of MDM2 is also active against mutant p53 myeloma

**Figure 3:**
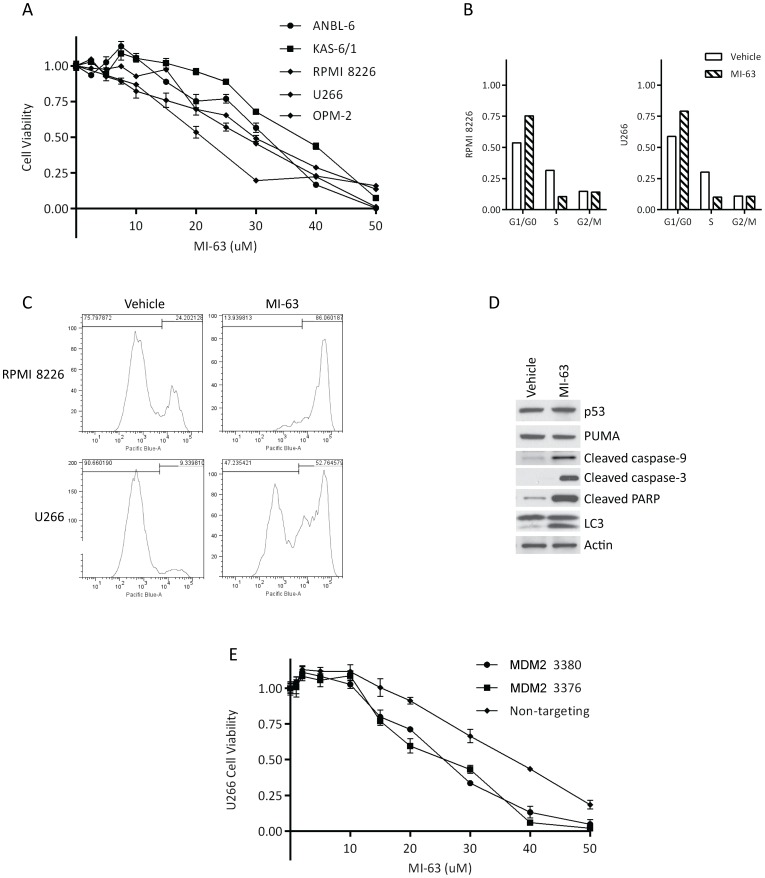
MI-63 is also active against mutant p53 myeloma cell lines. **A.** Cell viability assays were performed in a panel of mutant p53 myeloma cell lines, including ANBL-6, KAS-6/1, RPMI 8226, U266, and OPM-2 cells after exposure to MI-63 for 48 hours. **B.** Analysis of cell cycle distribution was performed in RPMI 8226 and U266 cells exposed to MI-63. **C.** Induction of apoptosis was evaluated by Annexin V staining and flow cytometry in RPMI 8226 and U266 cells treated with MI-63 at its IC_50_ for 48 hours. **D.** Extracts of RPMI 8226 cells treated with MI-63 were subjected to Western blotting to detect key intermediates in apoptosis and autophagy. **E.** The impact of MDM2 suppression on the efficacy of MI-63 was studied in U266 cells harboring either a control shRNA, or one of two different constructs that suppressed expression of MDM2.

The majority of newly-diagnosed patients with myeloma are felt to have wt p53, but the incidence of deletion at the 17p locus by fluorescence *in situ* hybridization ranges from 7%–11% [44], [45], and later increases to ∼22% in the relapsed and/or refractory setting [46]. We therefore sought to determine if MDM2 inhibitors could retain some activity in mutant (mut) p53 models using a different cell line panel (ANBL-6, KAS-6/1, RPMI 8226, U266, and OPM-2). While single µM concentrations were, in part as expected, largely ineffective, a dose-dependent decrease in viability was nonetheless seen in these mutant cells at higher concentrations (Figure 3A), with an IC_50_ in the range of 20–40 µM (ANBL-6: 30.1 µM; KAS-6/1: 36 µM; RPMI 8226: 27.9 µM; U266: 27.9 µM; OPM-2: 20.4 µM). Cell cycle analysis showed that RPMI 8226 and U266 cells exposed to MI-63 did accumulate at G_0_/G_1_ (Figure 3B). Furthermore, this was associated with induction of apoptosis as measured both by Annexin V staining (Figure 3C), and by activation of caspases-9 and -3, as well as cleavage of PARP (Figure 3D). This occurred without any impact on the expression levels of p53, or on downstream targets of wt p53, such as PUMA (Figure 3D), as would be expected in a mut p53 background. Given the high MI-63 concentrations required to induce cell death, we considered the possibility that this could be occurring through an off-target effect. U266 cells were therefore prepared that expressed either a non-targeting shRNA, or one of two different shRNAs that suppressed MDM2 (Figure S5). When these were exposed to MI-63, the two clones in which MDM2 was reduced showed a lower IC_50_ and therefore greater sensitivity compared to the controls (control shRNA IC_50_: 36.3 µM; MDM2 shRNA clone 3380 IC_50_: 24.7 µM; MDM2 shRNA clone 3376 IC_50_: 23.8 µM; Figure 3E). This finding is consistent with a continued role for MDM2 in the mechanism of action of MI-63 in these models, since decreased expression of MDM2 led to a need for less drug to elicit the same phenotype, whereas if MI-63 were active through a different target, MDM2 knockdown would not have changed the IC_50_.

### MDM2 inhibitors induce autophagy in mut p53 myeloma models

**Figure 4:**
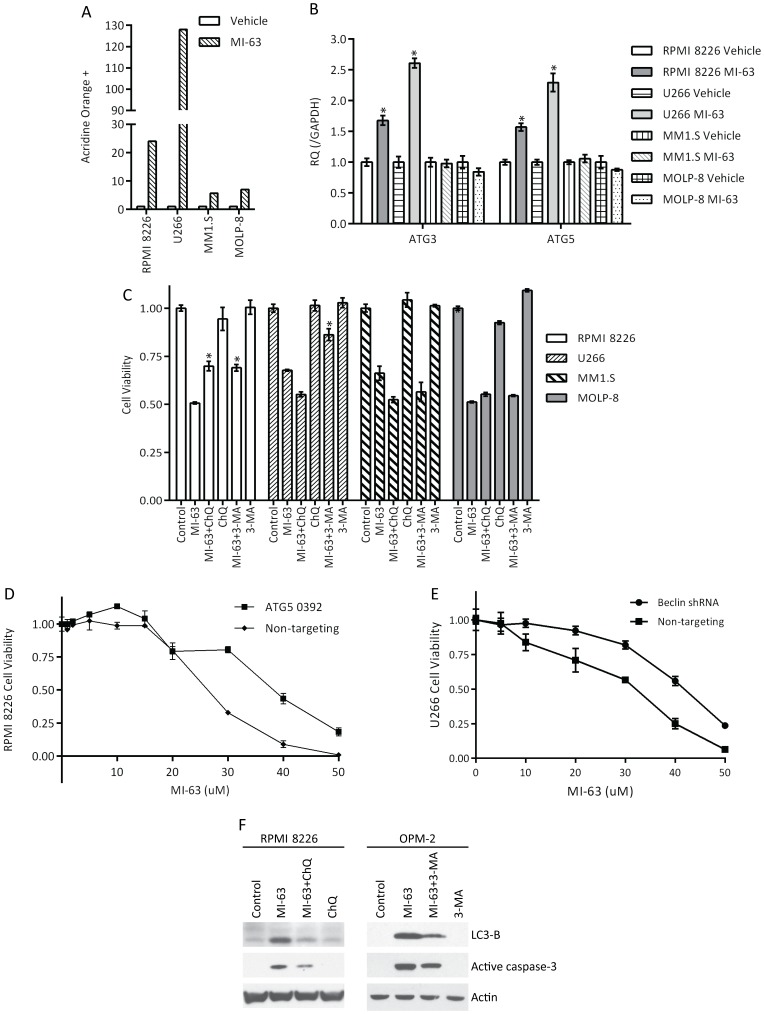
MI-63 induces autophagy in mutant p53 myeloma models. **A.** Acridine orange staining and flow cytometry were used to study the appearance of acidic vacuoles in mutant p53 RPMI 8226 and U266 cells, and wild-type p53 MM1.S and MOLP-8 cells exposed to the IC_50_ of MI-63 for 48 hours. **B.** Transcript levels of ATG3 and ATG5 were studied in mutant and wild-type p53 myeloma cells exposed to MI-63 (*p<0.005). **C.** The impact of the autophagy inhibitors chloroquine (100 nM) and 3-methyladenine (1 µM) on the activity of MI-63 against mutant and wild-type p53 cell lines was studied using viability assays (*p<0.005). **D.** ATG5 expression was suppressed with a Lentiviral shRNA in RPMI 8226 cells, and the effect of MI-63 was then studied in comparison to control shRNA cells. **E.** Beclin-1 was depleted with an shRNA, and the median inhibitory concentration of MI-63 was determined in comparison with a control shRNA. **F.** Activation of autophagy and apoptosis in mutant p53 myeloma cells treated with MI-63 and/or autophagy inhibitors.

While RPPA studies in wt p53 cells showed induction of type I apoptosis, RPMI 8226 and KAS6/1 cells exposed to MI-63 showed induction of several targets involved in autophagy, including AMP-activated protein kinase, and p70 S6 and S6 kinase (Figure S6 and results not shown). Moreover, by Western blotting, we did detect enhanced conversion of microtubule-associated protein (MAP)-1 light chain 3 (LC3) form I to form II (Figure 3D) after treatment with MI-63, which has been associated with autophagy. To obtain additional support for the possibility that autophagy was being induced, we stained cells with acridine orange, a hydrophobic dye that accumulates in acidic vesicles such as lysosomes and autophagic vacuoles. Compared to wt p53 MM1.S and MOLP-8 cells exposed to MI-63, which showed a slight increase in staining (Figure 4A), mut p53 RPMI 8226 and U266 cells showed a strong increase in acridine orange staining. The autophagic cell death program involves input from a number of genes, including ATG3 and 5, and we therefore performed qPCR to evaluate their expression. In wt p53 MM1.S and MOLP-8 cells exposed to MI-63, expression of ATG3 or 5 was unaltered (Figure 4B), but both were increased in mut p53 RPMI 8226 and U266 cells. Since autophagy may under some conditions promote cell survival, we evaluated the impact of MI-63 in the presence of chloroquine (ChQ), which raises lysosomal pH and inhibits autophagosome and lysosome fusion, or with 3-methyladenine (3-MA), an inhibitor of autophagic sequestration. In the context of wt p53, titrated ChQ slightly enhanced the cytotoxicity of MI-63, while 3-MA had no effect (Figure 4C). When mut p53 cells were studied, however, ChQ somewhat protected RPMI 8226 cells from MI-63, and 3-MA did so in both RPMI 8226 and U266 cells. Because these pharmacologic inhibitors can under some circumstances promote autophagy [47], we prepared RPMI 8226 cells harboring an shRNA to ATG5 (Figure S7A), and found that the IC_50_ to MI-63 was increased from 26.2 µM to 37.8 µM when ATG5 expression was suppressed (Figure 4D). Also, when the autophagy regulator Beclin-1 was suppressed (Figure S7B), the IC_50_ to MI-63 was again increased from 28.3 µM to 40.9 µM (Figure 4E). Finally, we evaluated the molecular effects of pharmacologic inhibitors of autophagy on apoptosis, and found that co-incubation of MI-63 with either ChQ or 3-MA reduced LC3 processing (Figure 4F), as expected, and also reduced the levels of activated caspase-3. Together, these findings show that MI-63 induces autophagy in mut p53 myeloma models, and suggests that there is cross-talk between autophagy and apoptosis in these plasma cells.

### MI-63 acts synergistically with other agents

Novel therapeutics for myeloma typically find their greatest efficacy in combination with standard, already approved agents, and we therefore sought to determine if MI-63 could show additive activity with other drugs. In MM1.S and MOLP-8 wt p53 cells, bortezomib alone and MI-63 alone showed single-agent activity, but the combinations showed additive to greater than additive effects in reducing myeloma cell viability (Figure S8A). Also, we tested the activity of a combination of MI-63 with the immunomodulatory agent lenalidomide, which does have direct anti-myeloma activity as a single agent *in vitro* [40]. As was the case for the combination with bortezomib, MI-63 with lenalidomide enhanced the reduction in viability induced by either agent alone (Figure S8B). Moreover, when MM1.S cells that had become resistant to lenalidomide [40], [41] were exposed to MI-63, this agent was able to overcome this resistance (Figure S8C).

**Figure 5:**
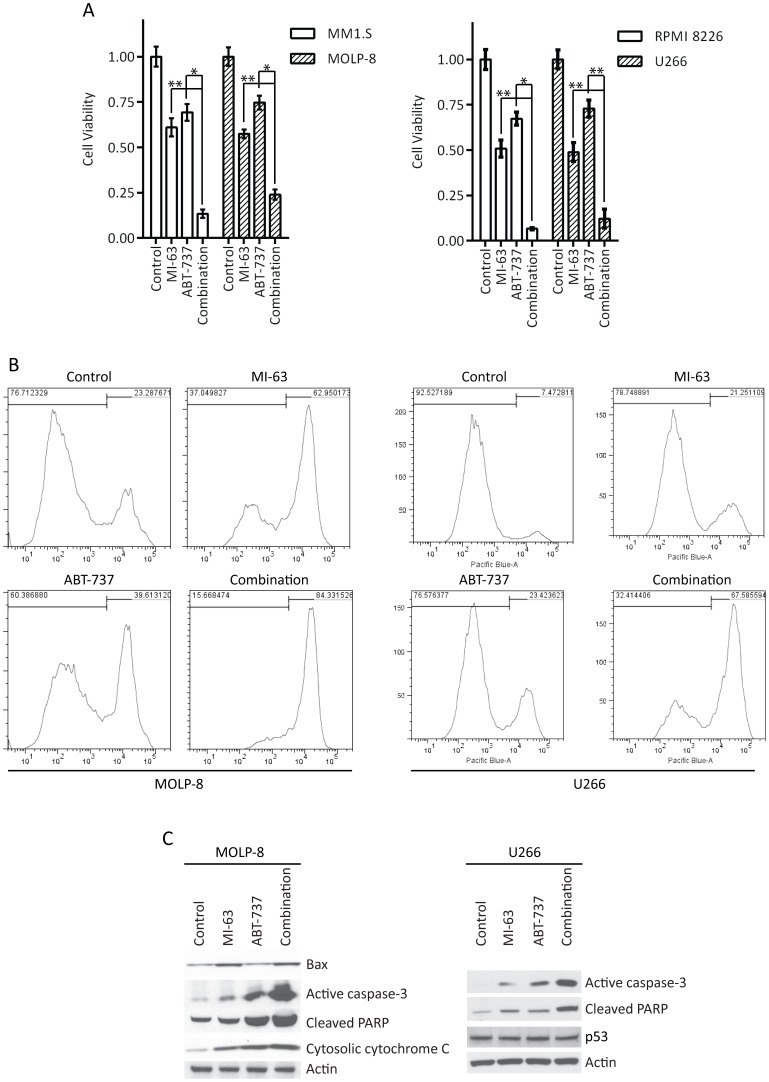
MI-63 acts synergistically with ABT-737. **A.** The combination of MI-63 and ABT-737 was studied in wild-type p53 MM1.S and MOLP-8 cells (left panel), and in mutant RPMI 8226 and U266 cells (right panel). Multiple doses of each drug were used for this experiment, with one representative condition shown. *p<0.005, ** *p*<0.05. **B.** Apoptosis was studied by staining for Annexin V in both wild-type p53 (MOLP-8; left panel) and mutant p53 (U266; right panel) cells treated with vehicle, MI-63, ABT-737, or the combination. **C.** Abundance of important intermediates in type I programmed cell death was studied in MOLP8 and U266 cells by Western blotting.

We also examined a combination of MI-63 with ABT-737, a BH3 mimetic inhibitor of B-cell lymphoma 2 (Bcl-2), Bcl-xL, and Bcl-w [48]. In wt p53 MM1.S and MOLP-8 cells, while both MI-63 and ABT-737 were able to reduce viability, the combination showed a greater effect than either agent alone (Figure 5A, left panel). Interestingly, this was also true in mut p53 RPMI 8226 and U266 cells (Figure 5A, right panel). By flow cytometry, the combination showed a strong increase in Annexin V staining in both MOLP-8 (Figure 5B, left panel) and U266 cells (Figure 5B, right panel), indicating that this was likely due to enhanced type I cell death, or apoptosis. Consistent with this possibility, Western blotting revealed increased activation of caspase-3 and cleavage of PARP in both MOLP-8 (Figure 5C, left panel) and U266 cells (Figure 5C, right panel).

**Figure 6:**
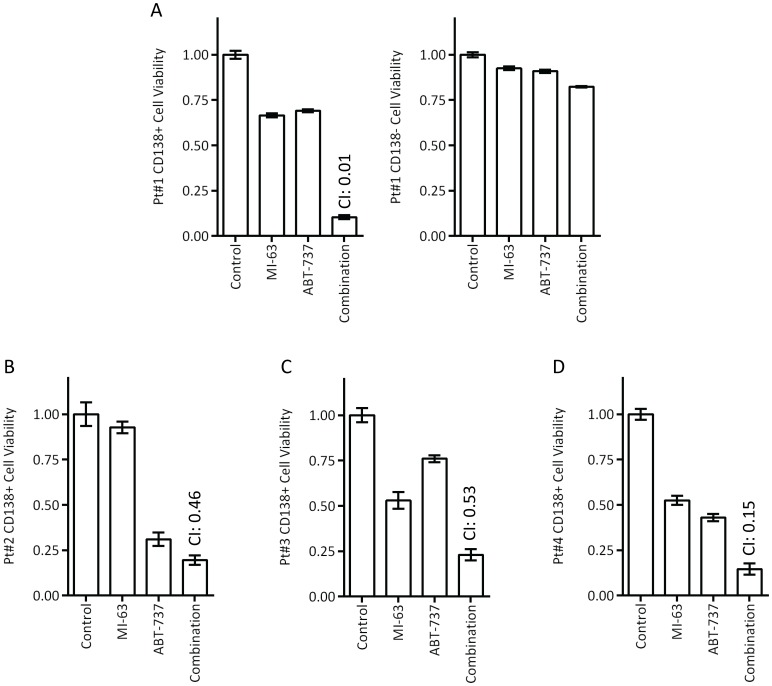
Combination studies in primary patient samples. **A.** The combination of MI-63 and ABT-737 was studied against CD138^+^ primary plasma cells from a patient with multiple myeloma (left panel), and CD138^−^ marrow cells (right panel). Note that multiple doses of each drug were used for this experiment to calculate combination indices, with one representative condition shown. **B–D.** Additional primary plasma cells isolated from unique patients were exposed to the combination of MI-63 and ABT-737.

Finally, we sought to examine the possibility that a combination of MI-63 and ABT-737 could show activity against primary patient samples, which were from bone marrow aspirates that were purified to obtain CD138^+^ plasma cells. In all four tested samples (Figure 6, A–D), both MI-63 and ABT-737 showed an ability to reduce cellular viability as single agents. When combined, the two drugs both revealed synergistic activity as measured by an increased reduction in viability compared with either agent by itself (combination index 0.01, 0.46, 0.53 and 0.15, respectively). Notably, CD138^−^ cells were also available from one patient (Figure 6A), and although some reduction in viability was seen with MI-63, ABT-737, and the combination, the magnitude was substantially less than that seen in CD138^+^ plasma cells, suggesting that there may be some measure of a therapeutic index. These data support possible translation to the clinic of the regimen of an MDM2 inhibitor and a BH3 mimetic.

## Discussion

Despite therapeutic advances that have improved the overall survival of myeloma patients, this plasma cell dyscrasia remains incurable, and is characterized clinically by multiple relapses, reduced benefit from subsequent treatments, and the development of refractory disease [4], [5], [6], [7], [8], [9]. These facts support the need for continued research to identify novel targets and treatment approaches that can be used alone, or in combination with current standards of care, first in the relapsed and/or refractory setting, and eventually perhaps as part of initial therapy. The current data provide further support for the possibility that agents targeting the MDM2 E3 ubiquitin ligase, such as MI-63, could form part of our armamentarium against myeloma. This agent was shown to be active against *in vitro* and *in vivo* wt p53 myeloma models through a p53-dependent program (Figure 1) including PUMA, Bax, p21, and Noxa, leading to cell cycle arrest and apoptosis (Figure 2). While MI-63 and the Nutlins have a similar mechanism of action, they differ in that Nutlins bind MDM2 residues Phe-19, Trp-23, and Leu-26 in the p53 interacting domain, while MI-63 binds these and also Leu-22 [34], [49], [50]. This suggests the possibility that MI-63 could prove to be more potent than is the case for the Nutlins. Interestingly, recent studies from our group showed that resistance mechanisms were similar for both agents in models of myeloma and mantle cell lymphoma, including formation of point mutations in the p53 DNA binding and dimerization domains [51].

While the potency of MI-63 was reduced against mut p53 models, cell death could still be induced using ten-fold higher drug concentrations, which was associated with cell cycle arrest and type I programmed cell death (Figure 3). Under these conditions, MDM2 inhibition in a mut p53 background seemed to be accompanied by activation of autophagy (Figure 4). Since inhibition of autophagy protected myeloma cells from MI-63, this at first appeared to suggest that autophagy could be contributing to cell death [52], [53]. However, additional studies revealed that the pharmacologic approaches which were used to inhibit autophagy also resulted in a reduction in type I cell death. These findings therefore argue in favor of cross-talk between these pathways [52], [53], such as perhaps at the level of Beclin-1 and Bcl-2. Interestingly, wt p53 itself can in some models induce autophagy, in part through damage-regulated autophagy modulator [54], [55], while in other models degradation of p53 through a pathway relying on MDM2 [56] induces autophagy. Additional studies will therefore be needed to determine the mechanism by which MDM2 inhibition leads to autophagy in a mut p53 background.

Rationally designed combination regimens often show increased activity against multiple myeloma compared with single agent approaches, and we were able to show that MI-63 did show enhanced efficacy when it was added to bortezomib (Figure S8). In this property, MI-63 is similar to Nutlin-3a, which has previously been shown to induce synergistic activity with bortezomib [31], [32] against *in vitro* myeloma models, largely in association with a p53-mediated cell death program. One difference is that Nutlin-3a was inhibited by adhesion-mediated drug resistance [31], whereas MI-63 showed similar activity in the absence or presence of human bone marrow-derived stromal cells (Figure 1), possibly suggesting it may be a superior clinical candidate. We were also able for the first time to show that MDM2 inhibition could act synergistically in combination with the immunomodulatory agent lenalidomide, and could also overcome lenalidomide resistance (Figure S8). The mechanisms of lenalidomide resistance are under active investigation, and while initial studies showed that mutations of Cereblon might be responsible [18], sequencing of primary samples has shown that this likely occurs only in a small minority [57]. Interestingly, while p21 abundance is enhanced by lenalidomide in drug-sensitive plasma cells, in drug-resistant cells that express low levels of p21, neither lenalidomide nor pomalidomide substantially enhance p21 expression [19]. It is therefore tempting to speculate that it is this restoration of p21 levels by inhibition of MDM2, leading to transactivation of p21 by p53, that is at the center of the ability of an MDM2 inhibitor to overcome lenalidomide resistance, at least in a p53 wt background. If MDM2 inhibitors are translated to the clinic as part of combination regimens with proteasome inhibitors or immunomodulatory drugs, attention may need to be paid to the potential for additive myelosuppression. This conclusion is based on results from a proof of concept study in patients with liposarcomas of single-agent RG-7112, an inhibitor in the Nutlin family, which induced neutropenia and thrombocytopenia in 30% and 40%, respectively, of patients [58].

Finally, we sought to determine if the addition of a BH3 mimetic could enhance the activity of an MDM2 inhibitor, in part with the rationale that the p53-mediated activation of Bax could be potentiated by blocking its interaction with Bcl-2. Consistent with this possibility, we did find that the combination of MI-63 and ABT-737 induced a greater reduction in cellular viability and induction of apoptosis than did either agent alone (Figure 5). This was accompanied by increased conversion of Bax and Bak to their active pro-apoptotic forms, as judged by conformation-specific antibodies (results not shown). Interestingly, the benefits of this combination may at least in part be due to possible off-target effects of MDM2 inhibitors. Indeed, the Nutlin class of drugs have been reported to also bind anti-apoptotic Bcl-2 family proteins, including Bcl-xL and Bcl-2 itself [59], [60], though whether this is also the case for MI-63 is not known. Notably, enhanced activity was seen even in the mut p53 models, which again seemed to occur through type I programmed cell death (Figure 5). Thus, while some combinations based on an MDM2 inhibitor may be expected to benefit only those patients with wt p53, such as with a death receptor 5 agonist [61], a regimen of a BH3 mimetic and an MDM2 inhibitor could benefit patients with either wt or mut p53. This would be especially important since recent studies with p53-specific probes and sequencing have suggested that higher rates of p53 deletion may be present than previously thought [62], [63], [64], particularly at the time of relapse [64]. Moreover, p53 deletion and mutation are universally recognized as poor prognostic features in myeloma, and approaches that could help such patients would be very welcome. We are therefore working at this time to translate such a regimen to the clinic in the relapsed and/or refractory setting.

## Supporting Information

Figure S1 **Impact of MI-63 on the interactions between MDM2 and other proteins.** MM1.S cells were treated with various doses of MI-63 for the indicated times, and cell extracts were subjected to immunoprecipitation with MDM2 antibodies, followed by Western blotting with antibodies specific to either p53 or MDM4.
(TIF)

Figure S2 **p53 knock down in MM1.S cells.** **A.** Impact of a Lentiviral-delivered shRNA targeting p53 compared to a control, non-targeting shRNA on p53 mRNA levels in MM1.S cells (*p<0.005). **B.** Western blotting shows the impact of these shRNAs on p53 protein expression.
(TIF)

Figure S3 **Impact of stromal cells on the efficacy of MI-63 in MOLP-8 cells.** The viability of MOLP-8 cells exposed to MI-63 either alone, or when propagated in co-culture with human-derived HS-5 stromal cells.
(TIF)

Figure S4 **Proteomic studies of wild-type p53 myeloma cells exposed to MI-63.** MM1.S and MOLP-8 cells were treated with MI-63 at its IC_50_ for 48 hours, and cell extracts were subjected to reverse phase protein array analysis. Changes are shown in selected proteins of interest. Error bars represent standard deviations of duplicate samples.
(TIF)

Figure S5 **Suppression of MDM2 in U266 myeloma cells.** U266 cell clones infected with one of two different shRNA constructs targeting MDM2 were isolated, and the reduction in MDM2 mRNA was evaluated by qPCR.
(TIF)

Figure S6 **Proteomic studies of RPMI 8226 cells exposed to MI-63.** RPMI 8226 cells were treated with MI-63 at its IC_50_ for 48 hours, and cell extracts were subjected to reverse phase protein array analysis. Changes are shown in selected proteins of interest. Error bars represent standard deviations of duplicate samples.
(TIF)

Figure S7 **Knockdown of ATG5 and Beclin-1.** **A.** Suppression of ATG5 in RPMI 8226 cells using a Lentiviral shRNA compared with a non-targeting control documented by Western blotting. **B.** Suppression of Beclin-1 in U266 cells using a Lentiviral shRNA compared with a non-targeting control documented by Western blotting.
(TIF)

Figure S8 **MI-63 augments the activity of other approved anti-myeloma agents.** **A.** MI-63 was combined with bortezomib in wild-type p53 MM1.S (left panel) and MOLP-8 (right panel) cells. Multiple doses of each drug were used with one representative condition shown (*p<0.005). **B.** MI-63 was combined with lenalidomide in wild-type p53 MM1.S (left panel) and MOLP-8 (right panel) cells. Multiple doses of each drug were used with one representative condition shown (*p<0.005). **C.** Cell viability was evaluated in lenalidomide-resistant MM1.S cells exposed to MI-63 for 48 hours, and compared to the efficacy of lenalidomide itself.
(TIF)
