# Supplementary figures and images for: Inhibition of the MDM2 E3 Ligase Induces Apoptosis and Autophagy in Wild-Type and Mutant p53 Models of Multiple Myeloma, and Acts Synergistically with ABT-737
Source: PLoS One. 2014 Sep 2;9(9):e103015. doi: 10.1371/journal.pone.0103015 (PMC4151993; doi:10.1371/journal.pone.0103015)

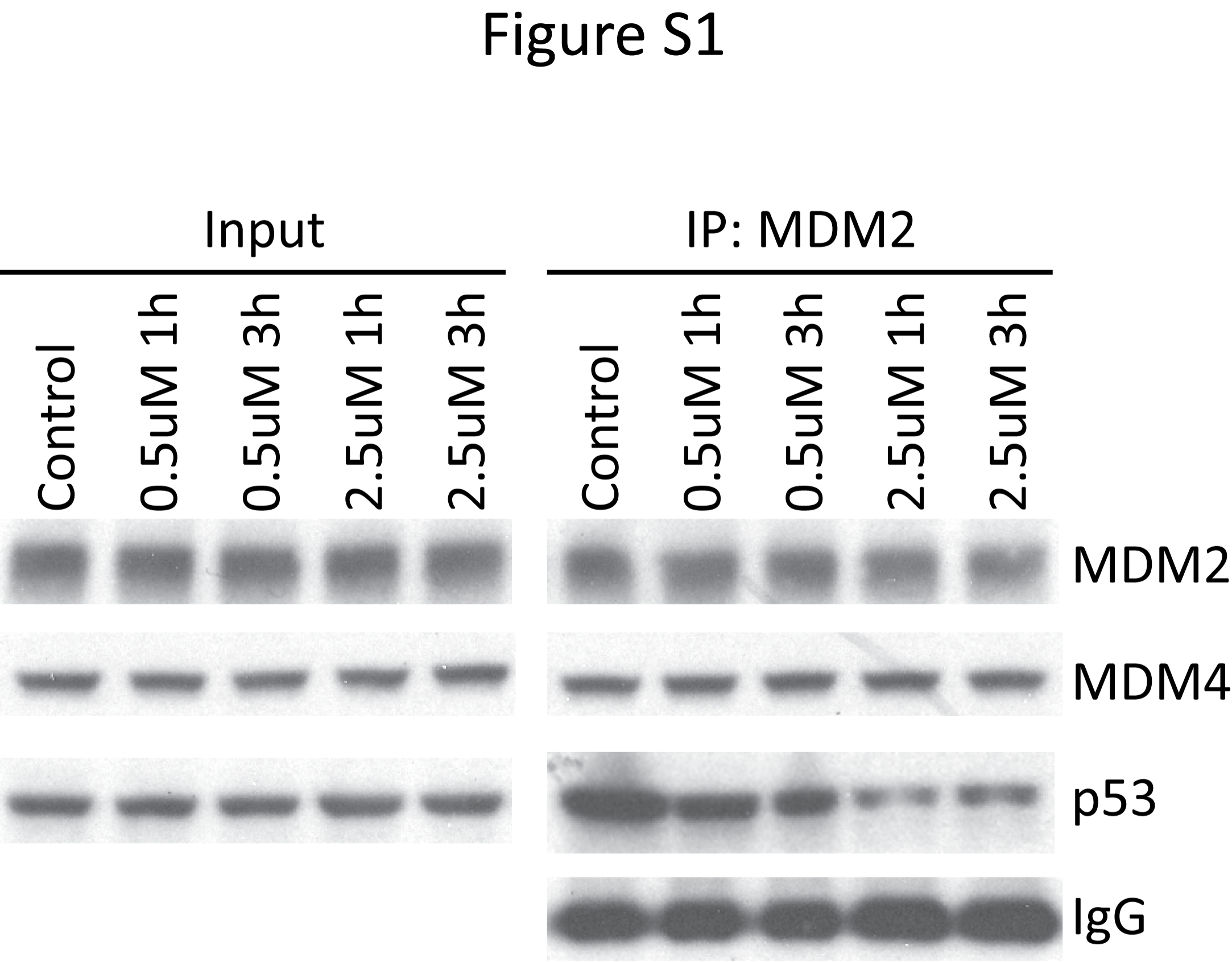

Supplement: Figure S1 — Impact of MI-63 on the interactions between MDM2 and other proteins. MM1.S cells were treated with various doses of MI-63 for the indicated times, and cell extracts were subjected to immunoprecipitation with MDM2 antibodies, followed by Western blotting with antibodies specific to either p53 or MDM4. (TIF) [file pone.0103015.s001.tif]

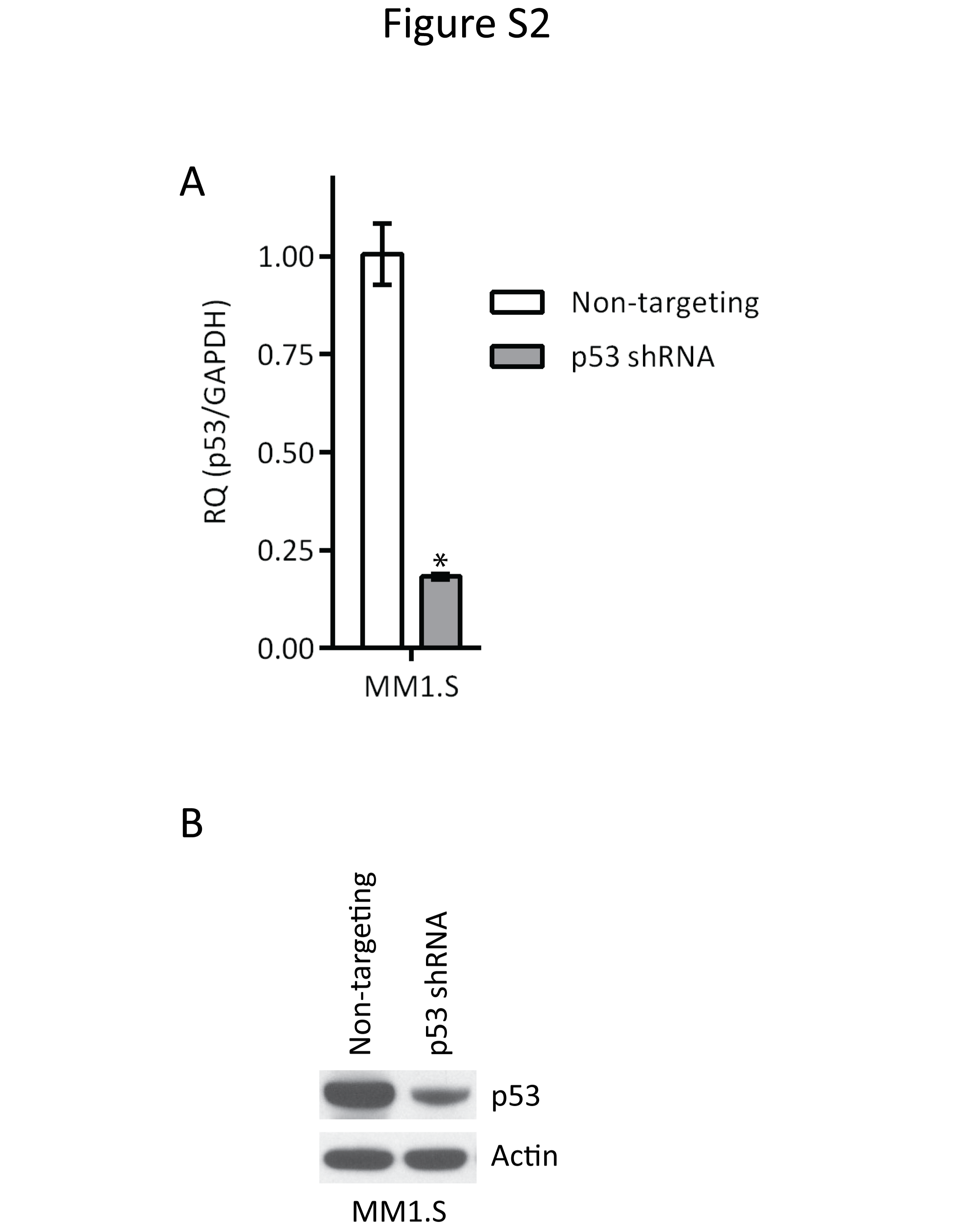

Supplement: Figure S2 — p53 knock down in MM1.S cells. A. Impact of a Lentiviral-delivered shRNA targeting p53 compared to a control, non-targeting shRNA on p53 mRNA levels in MM1.S cells (*p<0.005). B. Western blotting shows the impact of these shRNAs on p53 protein expression. (TIF) [file pone.0103015.s002.tif]

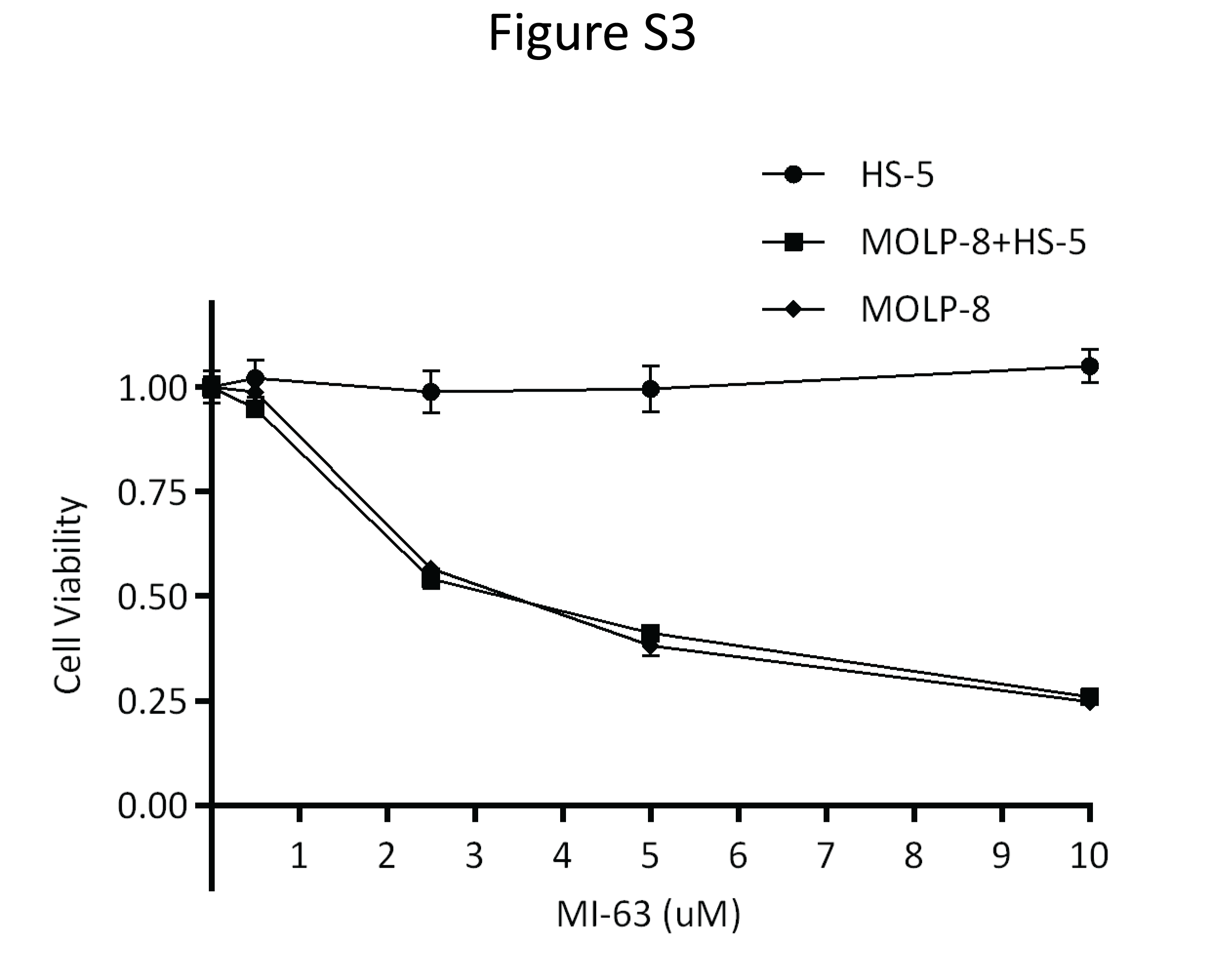

Supplement: Figure S3 — Impact of stromal cells on the efficacy of MI-63 in MOLP-8 cells. The viability of MOLP-8 cells exposed to MI-63 either alone, or when propagated in co-culture with human-derived HS-5 stromal cells. (TIF) [file pone.0103015.s003.tif]

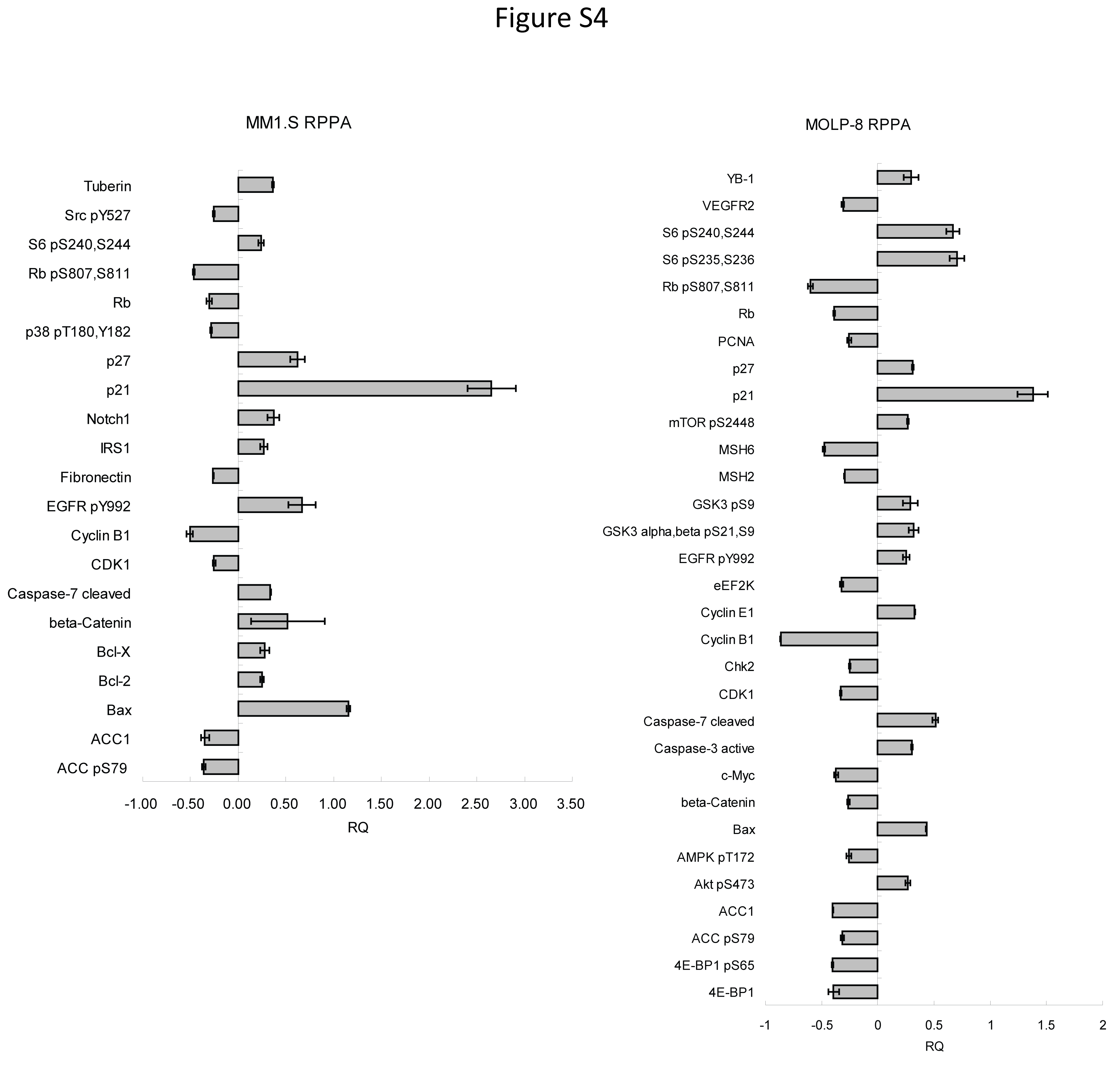

Supplement: Figure S4 — Proteomic studies of wild-type p53 myeloma cells exposed to MI-63. MM1.S and MOLP-8 cells were treated with MI-63 at its IC50 for 48 hours, and cell extracts were subjected to reverse phase protein array analysis. Changes are shown in selected proteins of interest. Error bars represent standard deviations of duplicate samples. (TIF) [file pone.0103015.s004.tif]

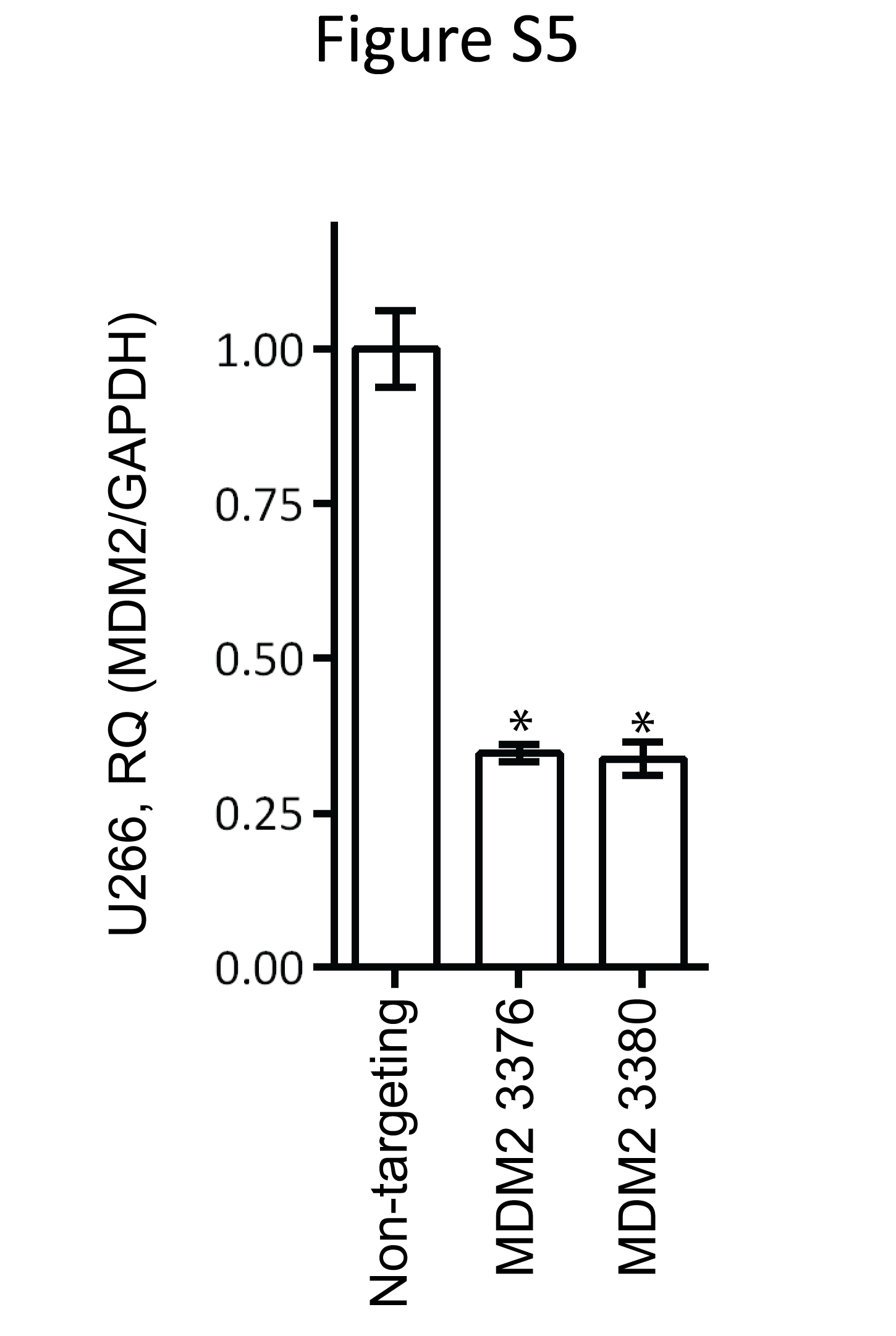

Supplement: Figure S5 — Suppression of MDM2 in U266 myeloma cells. U266 cell clones infected with one of two different shRNA constructs targeting MDM2 were isolated, and the reduction in MDM2 mRNA was evaluated by qPCR. (TIF) [file pone.0103015.s005.tif]

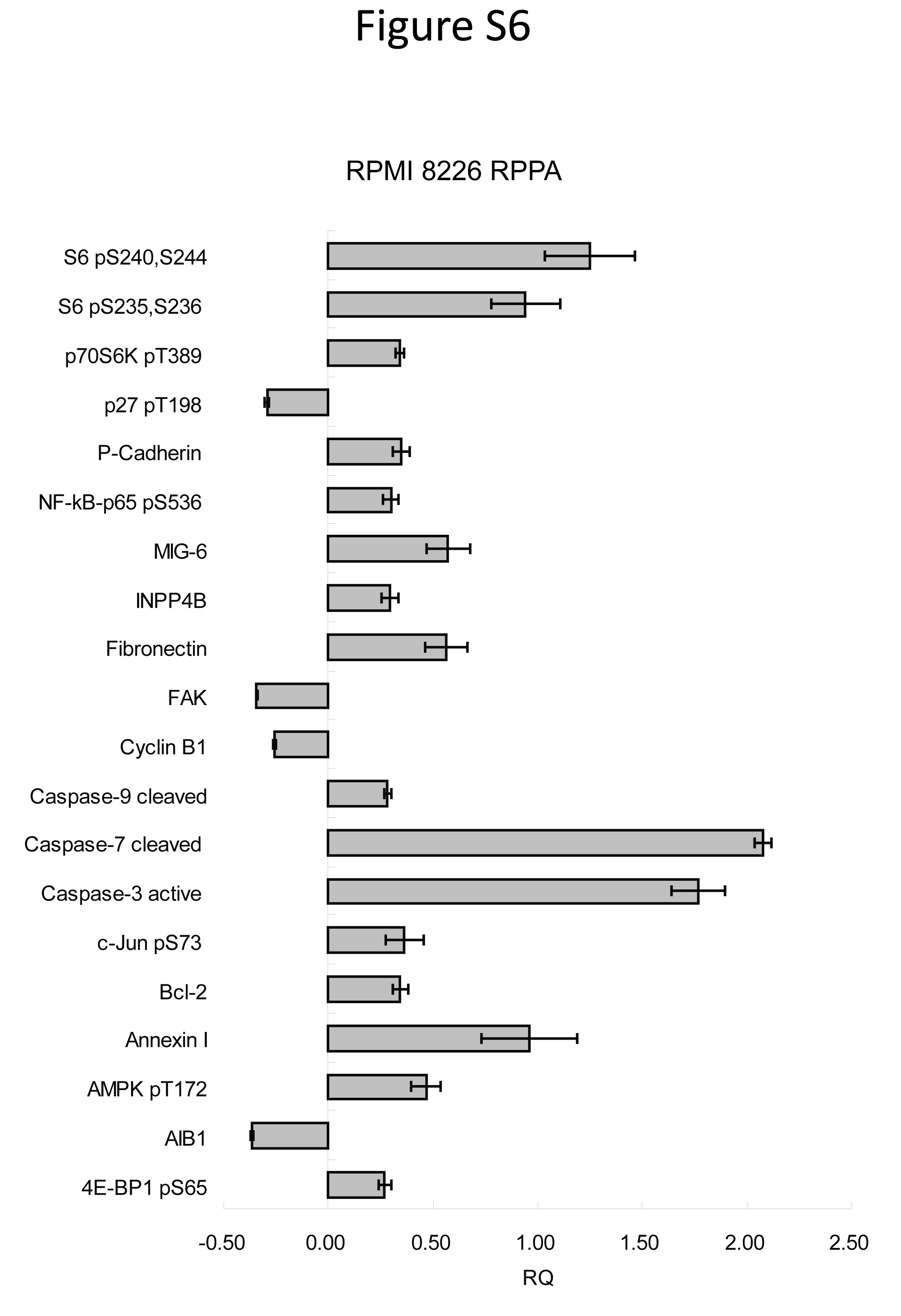

Supplement: Figure S6 — Proteomic studies of RPMI 8226 cells exposed to MI-63. RPMI 8226 cells were treated with MI-63 at its IC50 for 48 hours, and cell extracts were subjected to reverse phase protein array analysis. Changes are shown in selected proteins of interest. Error bars represent standard deviations of duplicate samples. (TIF) [file pone.0103015.s006.tif]

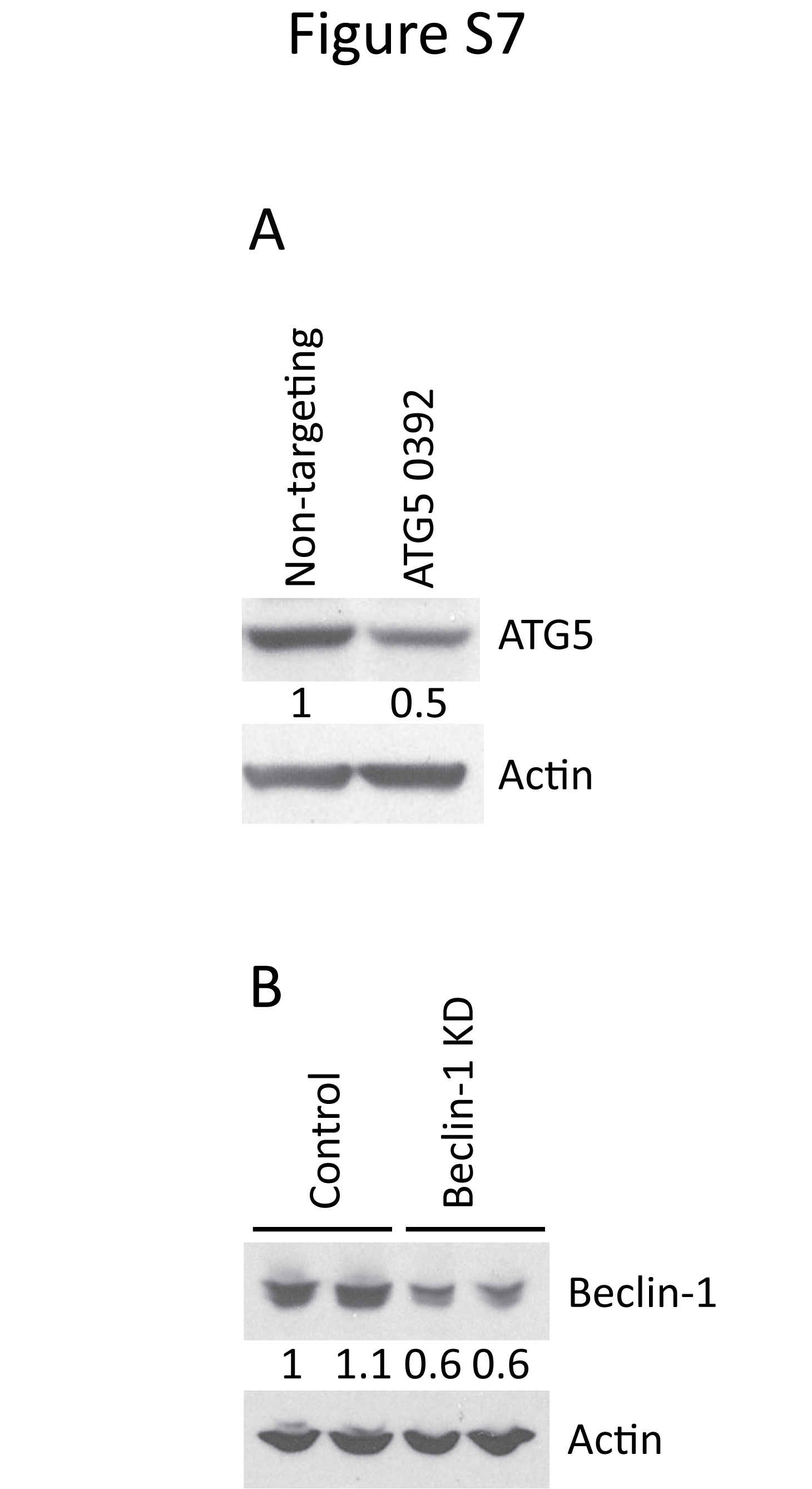

Supplement: Figure S7 — Knockdown of ATG5 and Beclin-1. A. Suppression of ATG5 in RPMI 8226 cells using a Lentiviral shRNA compared with a non-targeting control documented by Western blotting. B. Suppression of Beclin-1 in U266 cells using a Lentiviral shRNA compared with a non-targeting control documented by Western blotting. (TIF) [file pone.0103015.s007.tif]

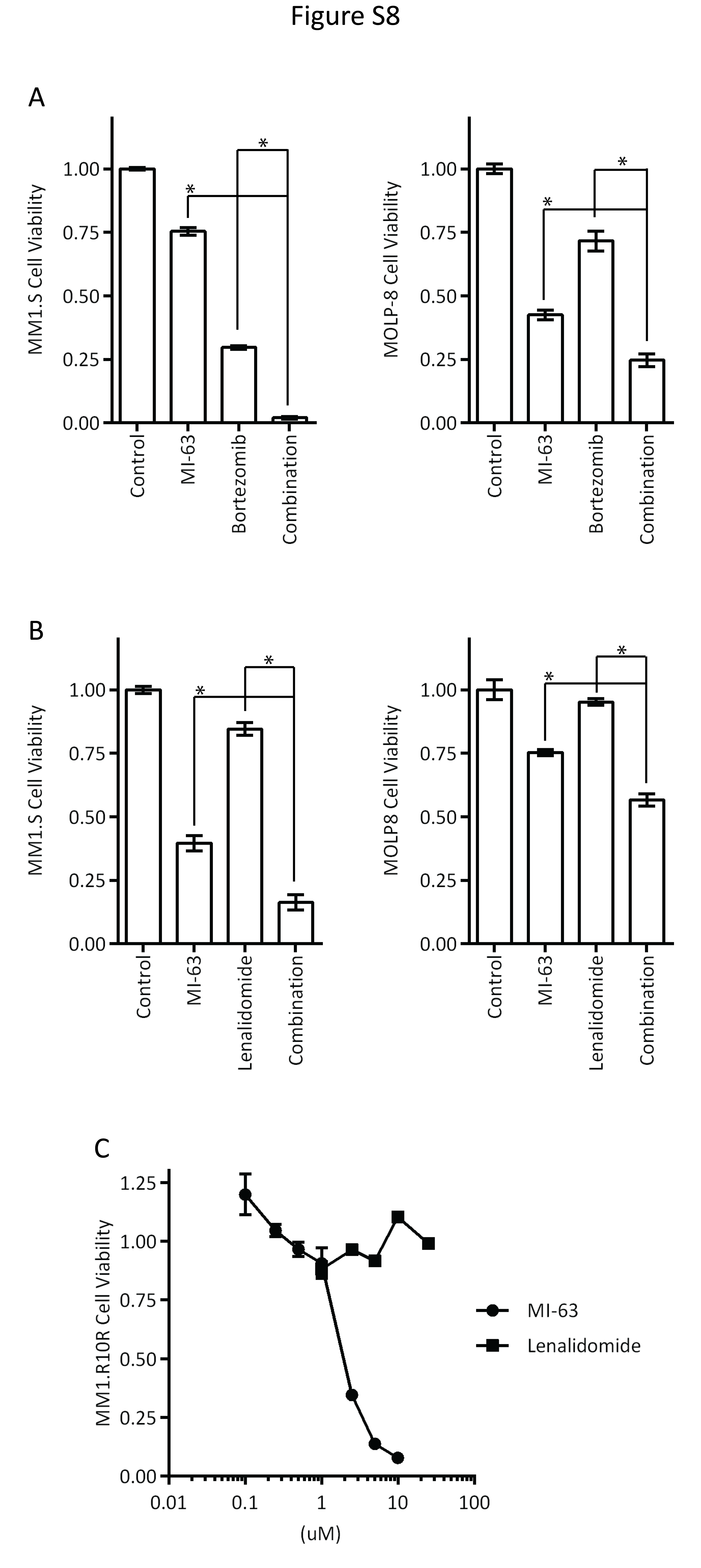

Supplement: Figure S8 — MI-63 augments the activity of other approved anti-myeloma agents. A. MI-63 was combined with bortezomib in wild-type p53 MM1.S (left panel) and MOLP-8 (right panel) cells. Multiple doses of each drug were used with one representative condition shown (*p<0.005). B. MI-63 was combined with lenalidomide in wild-type p53 MM1.S (left panel) and MOLP-8 (right panel) cells. Multiple doses of each drug were used with one representative condition shown (*p<0.005). C. Cell viability was evaluated in lenalidomide-resistant MM1.S cells exposed to MI-63 for 48 hours, and compared to the efficacy of lenalidomide itself. (TIF) [file pone.0103015.s008.tif]
